# Supplementary material for: Implementation strategies for telemental health: a systematic review
Source: BMC Health Serv Res. 2023 Jan 25;23:78. doi: 10.1186/s12913-022-08993-1 (PMC9873395; doi:10.1186/s12913-022-08993-1)
Supplement: Supplementary file 3 — Additional file 3: Appendix 3. Summary of implementation strategies and outcomes for all studies. [file 12913_2022_8993_MOESM3_ESM.docx]

Appendix 3: Summary of implementation strategies and outcomes for all studies

### Adler et al. (2013)

This low-quality study used two main categories of implementation strategies as part of a pilot project to improve the delivery of TMH in a Veterans Affairs (VA) service: ‘provide interactive assistance’ and ‘train and educate stakeholders’. Staff had monthly communication with therapists and met with clinical leaders every other month to discuss progress. Therapists completed online training and attended a video presentation by a psychotherapist with experience of TMH. Following the use of these strategies, the authors reported on three types of outcome relating to the TMH intervention: acceptability, feasibility, and sustainability. Acceptability outcomes varied between clinicians, with some reporting that TMH was not as difficult or disruptive as they had expected it to be, and that veteran acceptance of the approach surprised them. However, others reported little interest in conducting TMH. TMH was not viewed as feasible by all, with identified barriers to implementation including clinical demands, staff shortages, scheduling problems and equipment failures. TMH was not well sustained, as only two clinicians were offering TMH after 10 months. In many cases, clinical leaders had not acknowledged TMH as a priority.

### Baker-Ericzén et al. (2012)

This low-quality study used two types of implementation strategies in adopting a culturally adapted telemedicine intervention for Latina women with maternal depression. The first strategy used was ‘adapt and tailor to the context’, as the model used centrally located bilingual, bicultural Mexican American mental health advisors to adapt to the cultural context and address barriers. The second strategy was ‘develop stakeholder interrelationships’, as the model was also designed to facilitate communication between primary care and mental health services using a mental health advisor. These strategies were associated with high acceptability of the TMH intervention, as 97% of mothers reported overall satisfaction with the intervention and 100% rated the quality of the mental health advisor as high. Fidelity ratings of the intervention were also high, with a score of 83%.

### Chen et al. (2021)

In this descriptive-only study, the authors described the implementation of TMH psychology services at a VA TMH hub. Four categories of implementation strategy were used: ‘use evaluative and iterative strategies’, ‘adapt and tailor to the context’, ‘train and educate stakeholders’, and ‘support clinicians’. Quality improvement data was gathered to allow rapid identification of problems and adjustments to be made. Services were developed for TMH delivery based on a review of the literature and consultation with clinicians with previous experience of TMH. TMH was integrated into the existing psychology training programmes, with the goal of offering TMH training to all existing psychology training programmes within the next three years. Five new psychologists were hired for the main hub, weekly calls were set up between spoke sites and hub staff to establish the services. One staff member served as the primary point of contact for each spoke. These four strategies were associated with moderate to high adoption and penetration outcomes for the TMH intervention, as within five months the service reached its pre-established productivity goals of 80 veteran encounters per month, per provider for the first year. In the first nine months, from March 2017 to January 2018, 377 consults were received for TMH psychology services and 252 veterans engaged in TMH services. However, 32% did not receive treatment due to a variety of reasons, such as disengagement or discharge prior to TMH services being offered.

### Felker et al. (2021)

This medium-quality study described the development, implementation, and evaluation of a TMH training programme. Three strategies were used, the first of which was ‘provide interactive assistance’, in which training courses and workshops were conducted to address the specific practical aspects of providing TMH. Clinicians were encouraged to engage in TMH with at least two patients and attend at least ten one-hour consultation calls to ask questions related to TMH. Secondly, they used ‘adapt and tailor to the context’, in which internal facilitators from each team provided consultation to external facilitators regarding the unique clinical and cultural aspects of their team (e.g. patients served, types of services provided, administrative needs, technological needs). External and internal facilitators tailored the TMH training programme to address clinic specific culture and barriers and meet unique clinic goals. Finally, they used ‘train and educate stakeholders’, in which clinical champions and team leads supported training and implementation of TMH. Three outcomes relating to these strategies were reported. Clinicians viewed TMH as acceptable, as following the training, 95% of providers agreed (n=42) or strongly agreed (n=35) that they were satisﬁed with the training provided. Other implementation outcomes of these strategies were adoption, as providers reported increased knowledge, skills and interest in TMH after training, and appropriateness, as 95% of providers agreed (n=50) or strongly agreed (n=28) that the amount of information covered was sufficient to begin using TMH. 76% of participants agreed (n=45) or strongly agreed (n=17) that they felt conﬁdent using TMH after receiving training. Feasibility outcomes of the TMH intervention itself were also reported, with identified barriers to successful implementation following the above strategies including: lack of patient interest (45%), administrative burden (20%), preference for in-person appointments (18%), concern about increased workload (11%), not completed all of the training components (6%), lack of supervisor support (4%), lack of provider interest (4%), and other reasons (4%).

### Hensel et al. (2020)

This high-quality study reported on the barriers surrounding the use of telepsychiatry for emergency assessments and described an approach to overcoming those barriers for successful implementation of a programme to increase access to emergency psychiatric assessment. This study employed six different implementation strategies: ‘adapt and tailor to the context’ (an initial survey of barriers allowed the implementation to be tailored to address these), ‘develop stakeholder interrelationships’ (by using clinical champions and encouraging staff engagement), ‘train and educate stakeholders’, ‘engage consumers’ (clear explanations were given to patients and families regarding TMH), ‘utilize financial strategies’ (secured funding and reviewed the fee schedule), and ‘change infrastructure’ (installed dedicated equipment and made arrangements regarding existing equipment). Following these strategies, adoption (of the TMH intervention) implementation outcomes were reported, indicating successful adoption of TMH (for example, 243 assessments were completed and the percentage of transfers to other hospitals that were avoided increased from 0% pre-programme to 65% in December 2018).

### Lindsay et al. (2015)

This descriptive-only study reported outcomes of implementation of a video telehealth evidence-based psychotherapy programme for post-traumatic stress disorder and the results of a pilot facilitation strategy for implementation. Three implementation strategies were reported: ‘provide interactive assistance’, ‘adapt and tailor to the context’, and ‘train and educate stakeholders’. Technical support was provided through weekly consultation calls with a facilitator to discuss issues specific to the delivery of TMH. Site-specific implementation plans were tailored to the unique needs of the site, including needs of stakeholders. Intensive training in evidence-based practice for PTSD was also given to providers. One outcome directly related to these three strategies was reported, alongside one related to the TMH intervention. Clinicians reported a high degree of satisfaction and viewed the external facilitation model as very helpful in implementing video consultations (6.67 out of 7). They also found the regular facilitation calls to be very important in establishing video telehealth services. Penetration of the intervention was also reported: compared to baseline, participating sites averaged a 6.5-fold increase in psychotherapy sessions conducted via TMH, whereas non-participating sites only averaged a 1.7-fold increase.

### Lynch et al. (2020)

This medium-quality study used four different implementation strategies to examine the service utilisation of a complex psychosis (CP) and non-CP cohort attending a largely group-based recovery-oriented behavioural health service before and after conversion to TMH: ‘use evaluative and iterative strategies’, ‘provide interactive assistance’, ‘support clinicians’ and ‘engage consumers’.
In response to reports of problems with maintaining attention in virtual sessions, clinicians problem solved with clients to minimise distractions, used screen sharing features and interactive activities and provided additional brief breaks when needed. Virtual training on the features and functionality of telehealth platforms was provided to staff, factors to support and capture work from home productivity were considered for staff and individualized instruction regarding telehealth platforms was provided to service users as needed. Adoption and feasibility outcomes for the TMH intervention were good following the use of these strategies: for example, 90% of patients who were enrolled in the service agreed to telehealth sessions, and individualized treatment plans and group schedules were maintained following conversion to TMH.

### Lynch et al. (2021)

This medium-quality study explored factors which influenced successful conversion to telehealth in a group-based recovery orientated service. It reports the use of five different implementation strategies: ‘use evaluative and iterative strategies’, where the service responded to challenges identified by staff; ‘adapt and tailor to the context’, where group session material was adapted to be engaging on virtual platforms; ‘develop stakeholder interrelations’, with increased communication and support for staff; ‘engage consumers’, where, via a collaborative approach, some service users who found the use of TMH challenging helped the team develop web etiquette guidelines for other service users; and ‘change infrastructure’, facilitated by a “proactive culture at the clinic” when implementing TMH. Several implementation outcomes for the TMH intervention were reported following the use of these strategies: findings indicated high acceptability and adoption, TMH was viewed as appropriate for this patient group and fidelity of the group intervention was high. However, TMH was not viewed as feasible for everyone, as staff found it more challenging for clients who had technology or gaming addictions, or symptoms associated with attention deficit hyperactivity disorder or autism.

### Myers et al. (2020)

This medium-quality study used three strategies (‘provide interactive assistance’, ‘develop stakeholder interrelationships’ and ‘train and educate stakeholders’) during the implementation of VA Video Connect (a TMH platform). TMH champions assisted with enrolment into the system, procurement of equipment and completion of systems checks (for example, test calls, quality checks of audio and visual issues). In addition, site champions with previous experience supported implementation by assisting with mandatory training on policy and procedures and with developing selection criteria for determining appropriateness of treatment via TMH. Following the use of these strategies increased adoption of TMH was reported, with usage increasing by 42%. TMH was considered largely appropriate other than for suicidal or psychotic individuals, as providers expressed concerns about managing risk. There were also concerns around the feasibility of TMH for some service users, as it was deemed less suitable for those at ‘high risk’, those in crisis, and those without telephone or internet access. In terms of sustainability, the study concluded that this varied across sites depending on organisational constraints, such as administration time or other role commitments. Implementation cost was reported in relation to the use of site champions as a strategy, with the main cost being an increase in staff workload, as no additional funding was available for the role of site champion.

### Owens & Charles (2016)

This medium-quality study used two implementation strategies in investigating the use of a self-harming SMS text-messaging intervention (TeenTEXT) adapted for adolescents in child and adolescent mental health services (CAMHS). The first strategy was ‘use evaluative and iterative strategies’, in which clinicians and service users worked closely with the research team and software developers through a series of three iterations or feedback loops to optimise the intervention. The second strategy was to ‘develop stakeholder interrelations’, in which three clinicians in each team supported and mentored each other for the duration of the study and cascaded knowledge through the team, influencing others to adopt the intervention. Implementation outcomes following the use of these strategies were mixed: although clinicians viewed the intervention as acceptable, barriers to adoption and feasibility of the TMH intervention were identified as CAMHS teams reported being under high pressure before implementation, which limited their time to learn the intervention. The intervention was not viewed as appropriate for a CAMHS setting as clinicians saw only the most acute and complex cases and duration of contact with CAMHS is typically short.

### Puspitasari et al. (2021a)

This medium-quality study used two strategies to promote implementation of a group-based transitional day programme for adults with transdiagnostic conditions at risk of psychiatric hospitalization. Firstly, the strategy ‘train and educate stakeholders’ was used, with counsellors attending weekly consultation meetings facilitated by a clinical psychologist to ensure treatment adherence and fidelity. Secondly, they employed the strategy ‘engage consumers’, in which service users who were accepted into the programme received assistance from programme staff and information technology support staff to prepare for the first TMH session. An additional counsellor was on hand to assist patients with any technological issues during group sessions. Two implementation outcomes relating to the TMH intervention were investigated following the deployment of these strategies: adoption and feasibility. The completion rate of the programme was 70/76, which was higher than typical completion rates for psychiatric intensive outpatient or partial hospitalization programmes. The use of several Zoom features, including chat, whiteboard, screen sharing and waiting room, improved feasibility. It was also feasible to conduct psychotherapy experiential exercises, such as mindfulness, via videoconferencing.

### Puspitasari et al. (2021b)

This descriptive study reported on the use of five implementation strategies for the rapid adoption and implementation of teletherapy due to the COVID-19 pandemic in an intensive outpatient programme for adults with severe mental illness. These were: ‘use evaluative and iterative strategies’, ‘provide interactive assistance’, ‘adapt and tailor to the context’, ‘develop stakeholder interrelationships’, and ‘train and educate stakeholders’. The overarching approach was to involve a multi-disciplinary TMH committee who coordinated the change to TMH by providing training, ensuring clinicians had access to necessary technology and IT support, reviewing and expanding guidelines and policies, identifying TMH champions and providing ongoing support and supervision. Following the deployment of these strategies three outcomes relating to the TMH intervention were measured: adoption, feasibility and penetration. The study identified education, training, and ongoing supervision as being particularly important in increasing adoption and engaging clinicians. Data on patient attrition indicated that TMH is feasible to assure patient retention, since many service users completed the programme and the average number of sessions attended was high. A plan had also been established by the pilot site to initiate full implementation following pilot implementation, indicating high penetration.

### Sharma et al. (2020)

This descriptive study used five implementation strategies in investigating the implementation components involved in transitioning a comprehensive outpatient child and adolescent psychiatry programme to a home-based TMH virtual clinic. These were: ‘use evaluative and iterative strategies’, ‘provide interactive assistance’, ‘adapt and tailor to the context’, ‘train and educate stakeholders’, and ‘engage consumers’. For example, pilot tests were conducted with three small groups of parents, technical guidance was provided to all clinicians after group TMH training sessions, and a “cheat sheet” was developed for families to help them access TMH. Each day the clinic analysed and adapted to the latest government rules regarding stay-at-home mandates, and to patient and staff needs. If a family was not able to participate in the intervention due to lack of internet access, then a phone appointment was offered to ensure equity. These strategies resulted in five outcomes relating to the TMH intervention. Adoption of TMH was delayed by failures of the videoconferencing platform, however penetration of telemental heath was overall successful, as by April 10^th^ 2020, all established outpatients were offered remote appointments. Findings also indicated that these strategies meant it was feasible to rapidly expand the existing telehealth infrastructure during an emergency. In terms of implementation cost, the service reported that less funding was generated from interim phone appointments (before proper TMH could take place) than from face-to-face or TMH appointments. The study authors viewed TMH as sustainable and as having the potential to help overcome barriers to treatment, such as distance, transportation and scheduling.

### Taylor et al. (2019)

This high-quality study used two strategies - ‘use evaluative and iterative strategies’ and ‘develop stakeholder interrelationships’ - to investigate the importance of clinical facilitation for the implementation and sustainability of TMH in perinatal and infant mental health services. Firstly, a pilot project established the efficacy of the intervention in improving the skills and knowledge of local health service providers but identified a need for additional clinical support in specialist areas, which was therefore integrated into the model. As a result of a pilot project, General Practitioners (GPs), mental health professionals and other service providers were offered access to secondary care consultations with perinatal and infant psychiatrists. The service also employed a clinical facilitator who was responsible for service promotion, site visits, staff education and training, co-ordinating case conferences and video consultations. Two outcomes relating to the TMH intervention were reported following the deployment of these implementation strategies: acceptability and appropriateness. All mental health workers who had used TMH evaluated it positively, reporting that it allowed expert input into care planning, reduced professional isolation, upskilled remote workers and provided a sense of security for remote care providers. Regarding appropriateness, the study showed that TMH can help address unmet need for specialist mental health services in regional, rural and remote areas. In terms of the implementation strategies themselves, the study concluded that ongoing clinical facilitation is necessary for the sustainability of TMH services due to intermittent demand and local impediments, such as fragmentation of service providers and transiency of the workforce, which can make continued unsupported use of new technology challenging.
